# Chronic treatment with fluoride affects the jejunum: insights from proteomics and enteric innervation analysis

**DOI:** 10.1038/s41598-018-21533-4

**Published:** 2018-02-16

**Authors:** Aline Salgado Dionizio, Carina Guimarães Souza Melo, Isabela Tomazini Sabino-Arias, Talita Mendes Silva Ventura, Aline Lima Leite, Sara Raquel Garcia Souza, Erika Xavier Santos, Alessandro Domingues Heubel, Juliana Gadelha Souza, Juliana Vanessa Colombo Martins Perles, Jacqueline Nelisis Zanoni, Marília Afonso Rabelo Buzalaf

**Affiliations:** 1 Department of Biological Sciences, Bauru School of Dentistry, University of São Paulo, Bauru, Brazil; 2 Department of Morphophysiological Sciences, State University of Maringá, Maringá, Brazil

## Abstract

Gastrointestinal symptoms are the first signs of fluoride (F) toxicity. In the present study, the jejunum of rats chronically exposed to F was evaluated by proteomics, as well as by morphological analysis. *Wistar* rats received water containing 0, 10 or 50 mgF/L during 30 days. HuC/D, neuronal Nitric Oxide (nNOS), Vasoactive Intestinal Peptide (VIP), Calcitonin Gene Related Peptide (CGRP), and Substance P (SP) were detected in the myenteric plexus of the jejunum by immunofluorescence. The density of nNOS-IR neurons was significantly decreased (compared to both control and 10 mgF/L groups), while the VIP-IR varicosities were significantly increased (compared to control) in the group treated with the highest F concentration. Significant morphological changes were seen observed in the density of HUC/D-IR neurons and in the area of SP-IR varicosities for F-treated groups compared to control. Changes in the abundance of various proteins correlated with relevant biological processes, such as protein synthesis, glucose homeostasis and energy metabolism were revealed by proteomics.

## Introduction

Fluoride (F) is considered one of the essential elements for the maintenance of the normal cellular processes in the organism^1^ and is largely employed in dentistry to control dental caries^2^. However, when excessive amounts are ingested, F can induce oxidative stress and lipid peroxidation, alter intracellular homeostasis and cell cycle, disrupt communication between cells and signal transduction and induce apoptosis^3^.

Nearly 25% of ingested F is absorbed from the stomach as an undissociated molecule (HF) in a process that is inversely related to pH^4^, while the remainder is absorbed in the ionic form (F^−^) from the small intestine, in a pH-independent manner^5^. Due to its major role in F absorption, the gastrointestinal tract (GIT) is regarded as the principal way of exposure to F^6^. Thus, gastrointestinal symptoms, including motion sickness, vomiting, diarrhea and abdominal pain are the first signs of F toxicity^7–10^.

The Enteric Nervous System (ENS) is an interlinked network of neurons disposed in the intestinal walls that controls the function of the GIT^11^. Due to its control function, changes in ENS affect the absorption, secretion, permeability and motility of the GIT^12^. Recently, immunofluorescence techniques revealed important alterations in the morphology of different types of enteric neurons and proteomic analysis demonstrated changes in the expression of several proteins of the duodenum of rats^13^ after chronic exposure to F, providing the first insights for the comprehension of the mechanisms underlying the actions of F on the bowel. However, the effect of F on the ENS and proteomic profile of the jejunum has never been reported. Considering that each segment of the small intestine has distinct anatomical, histological and physiological characteristics with functional implications^14^, this study evaluated the morphology of distinct subtypes of enteric neurons of the jejunum after chronic exposure to F. Quantitative label-free proteomics tools were employed to evaluate the changes on the pattern of protein profile of the jejunum, after exposure to F, in attempt to provide mechanistic explanations for the effects of this ion in the intestine.

## Material and Methods

### Animals and treatment

The Ethics Committee for Animal Experiments of Bauru Dental School, University of São Paulo approved all experimental protocols (#014/2011 and #012/2016). All experimental protocols were approved by. The assays conformed with the guidelines of the National Research Council. Eighteen adult male rats (60 days of life - *Rattus norvegicus*, Wistar type) were randomly assigned to 3 groups (n = 6/group). They remained one by one in metabolic cages, having access to water and food *ad libitum* under standard conditions of light and temperature. The animals received deionized water (0 mgF/L), 10 mgF/L and 50 mgF/L for 30 days as sodium fluoride (NaF) dissolved in deionized water, in order to simulate chronic intoxication with F. Since rodents metabolize F 5 times faster than humans, these F concentrations correspond to ~2 and 10 mg/L in the drinking water of humans^15^. After the experimental period, the animals received an intramuscular injection of anesthetic and muscle relaxant (ketamine chlorhydrate and xylazine chlorhydrate, respectively). While the rats were anesthetized, the peritoneal and thoracic cavities were exposed, and the heart was punctured for blood collection, using a heparinized syringe. Plasma was obtained by centrifugation at 800 g for 5 minutes for quantification of F, described in a previous publication^13^. After blood collection, the jejunum was collected for histological, immunofluorescence and proteomic analysis. For the collection of the jejunum, animal chow was removed from the animals 18 hours prior the euthanasia to decrease the volume of fecal material inside the small intestine, facilitating the cleaning process for posterior processing. After laparotomy, to remove the jejunum, initially the small intestine was localized, and the jejunum proximal limit was identified by the portion after the duodenojejunal flexure that is attached to the posterior abdominal wall by the ligament of Treitz. After incisions in the flexure and ligament, 20 centimeters distally to the incision were despised and then 15 centimeters of the jejunum were harvested for processing. After harvesting, the jejunum was washed with PBS solution applied several times with a syringe in the lumen to remove completely any residue of fecal material.

### Histological analysis

This analysis was performed exactly as described by Melo, *et al*.^13^.

### Myenteric plexus immunohistochemistry, morphometric and semi-quantitative analysis

These analyses were performed exactly as described by Melo, *et al*.^13^.

### Proteomic analysis

The frozen jejunum was homogenized in a cryogenic mill (model 6770, SPEX, Metuchen, NJ, EUA). Samples from 2 animals were pooled and analyses were carried out in triplicates. Protein extraction was performed by incubation in lysis buffer (7 M urea, 2 M thiourea, 40 mM DTT, all diluted in AMBIC solution) under constant stirring at 4 °C. Samples were centrifuged at 14000 rpm for 30 min at 4 °C and the supernatant was collected. Protein quantification was performed^16^. To 50 μL of each sample (containing 50 μg protein) 25 μL of 0.2% Rapigest (WATERS cat#186001861) was added, followed by agitation and then 10 μL 50 mM AMBIC were added. Samples were incubated for 30 min at 37 °C. Samples were reduced (2.5 μL 100 mM DTT; BIORAD, cat# 161–0611) and alkylated (2.5 μL 300 mM IAA; GE, cat# RPN 6302 V) under dark at room temperature for 30 min. Digestion was performed at 37 °C overnight by adding 100 ng trypsin (PROMEGA, cat #V5280). After digestion, 10 µL of 5% TFA were added, incubated for 90 min at 37 °C and sample was centrifuged (14000 rpm at 6 °C for 30 min). Supernatant was purified using C 18 Spin columns (PIERCE, cat #89870). Samples were resuspended in 200 μL 3% acetonitrile.

### LC-MS/MS and bioinformatics analyses

The peptides identification was done on a nanoAcquity UPLC-Xevo QTof MS system (WATERS, Manchester, UK), using the PLGS software, as previously described^17^. Difference in abundance among the groups was obtained using the Monte-Carlo algorithm in the ProteinLynx Global Server (PLGS) software and displayed as p < 0.05 for down-regulated proteins and 1 − p > 0.95 for up-regulated proteins. Bioinformatics analysis was done to compare the treated groups with the control group (Tables S1–S5), as previously reported^17–20^. The software CYTOSCAPE 3.0.4 (JAVA) was used to build networks of molecular interaction between the identified proteins, with the aid of ClueGo and ClusterMarker applications.

## Results

### Morphological analysis of the jejunum wall thickness

The mean (±SD) thickness of the jejunum tunica muscularis was significantly higher in the 50 mgF/L (93.0 ± 1.4 µm^2^) when compared to control (81.5 ± 1.1 µm^2^) and 10 mgF/L (84.2 ± 2.5 µm^2^) groups (Bonferroni’s test, p < 0.05). The total thickness of the jejunum wall was significantly lower in the 50 mgF/L (742.25 ± 7.8 µm^2^) and 10 mgF/L (734.4 ± 11.8 µm^2^) when compared to control (783.15 ± 5.8 µm^2^) (Bonferroni’s test, p < 0.05).

### Myenteric HuC/D – IR neurons analysis

When the general population of neuron was morphometrically analyzed, the cell bodies areas (µm^2^) of the HuC/D–IR neurons were not significantly different among the groups (p > 0.05). In the semi-quantitative analyses (neurons/cm^2^), a significant decrease in the density was observed in the treated groups when compared with control (p < 0.05) (Table 1).

**Table 1:** Means and standard errors of the values of the cell bodies areas and density of HUC/D-IR and nNOS-IR neurons and VIP-IR, CGRP-IR, and SP-IR values of myenteric neurons varicosities areas of the jejunum of rats chronically exposed or not to fluoride in the drinking water.

ANALYSIS	Control	10 mgF/L	50 mgF/L
Cell bodies areas of the HuC/D-IR neurons (µm^2^)	304.9 ± 3.5^a^	310.7 ± 3.8^a^	304.8 ± 3.8^a^
Density HuC/D-IR neurons (neurons/cm^2^)	16,968 ± 350^a^	15,420 ± 392^b^	15,230 ± 380^b^
Cell bodies areas of the nNOS-IR neurons (µm^2^)	291.4 ± 3.2^a^	296.6 ± 3.5^a^	289.6 ± 2.9^a^
Density nNOS-IR neurons (neurons/cm^2^)	5,725 ± 123^a^	5,559 ± 134^a^	5,176 ± 146^b^
Area VIP-IR varicosities (µm^2^)	3.08 ± 0.52^a^	3.98 ± 0.03^ab^	4.46 ± 0.04^b^
Area CGRP-IR varicosities (µm^2^)	3.31 ± 0.03^a^	3.35 ± 0.04^a^	3.38 ± 0.03^a^
Area SP-IR varicosities (µm^2^)	2.81 ± 0.01^a^	4.86 ± 0.03^b^	4.64 ± 0.03^c^

Means followed by different letters in the same line are significantly different according to Fisher’s test (density HuC/D-IR and nNOS-IR neurons) or Tukey’s test (other variables). p < 0.05. n = 6.

### Myenteric nNOS –IR neurons analysis

The cell bodies areas (µm^2^) of the nNOS-IR neurons did not present a significant difference among the groups (p > 0.05) in the morphometric analysis. As for the semi-quantitative analyses, a decrease in the mean value of the density for the group treated with 50 mgF/L when compared with the other groups was observed (p < 0.05; Table 1).

### Myenteric varicosities VIP-IR, CGRP-IR or SP-IR morphometric analysis

A significant increase in the VIP-IR varicosity areas (µm^2^) was detected in the group treated with 50 mgF/L when compared with the control group (p < 0.05). For the CGRP-IR varicosity areas, the groups did not differ significantly (p > 0.05). However, SP-IP varicosity areas were significantly increased in the treated groups when compared with control. In addition, the group treated with 10mgF/L presented an area significantly higher than the group treated with 50 mgF/L (Table 1).

**Figure 1:**
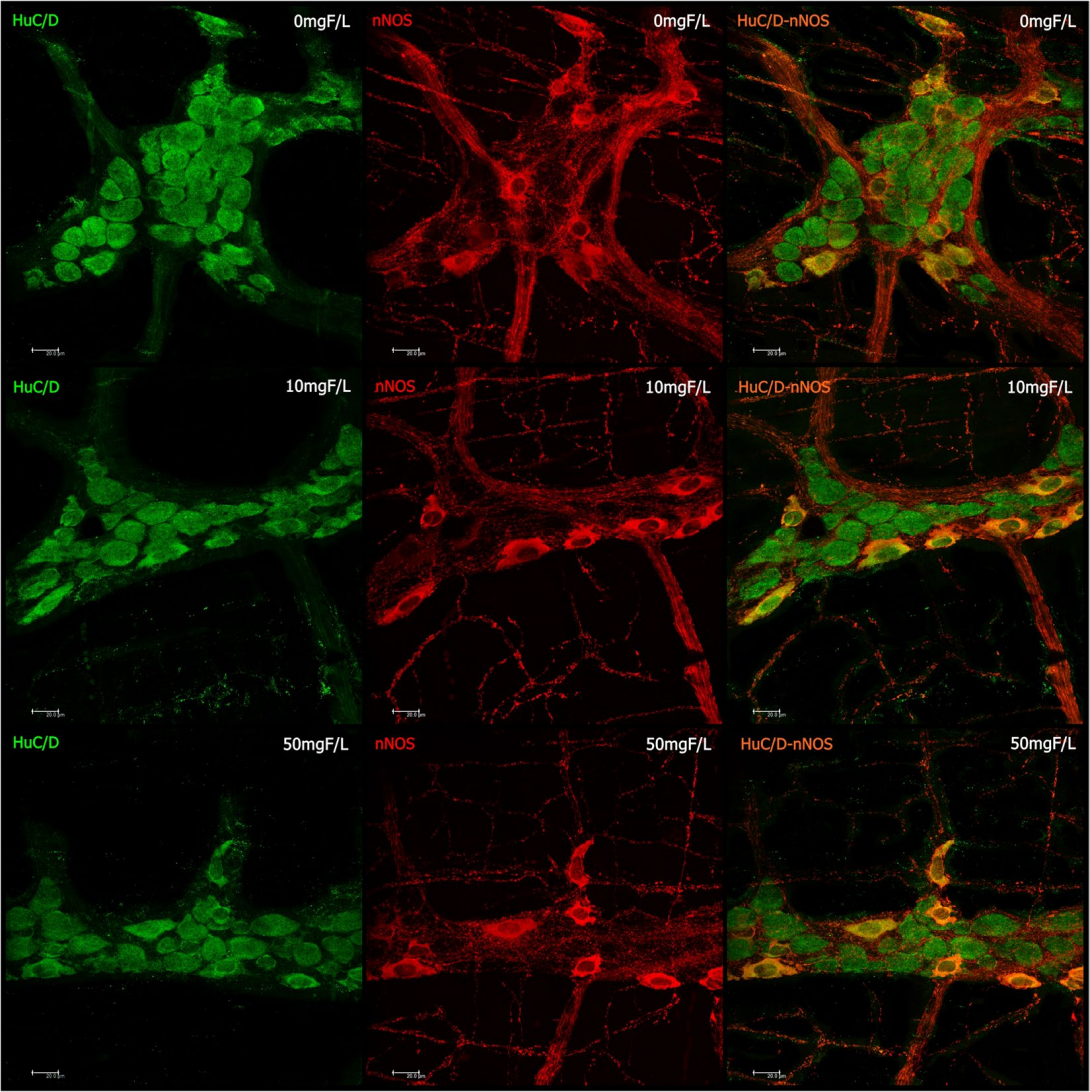
Photomicrography of myenteric neurons of the rats jejunum stained for HuC/D (green), nNOS (red), and double-labeling (HuC/D and nNOS) for the control group (0 mgF/L) and for the groups treated with 10 and 50 mgF/L. 20x Objective.

**Figure 2:**
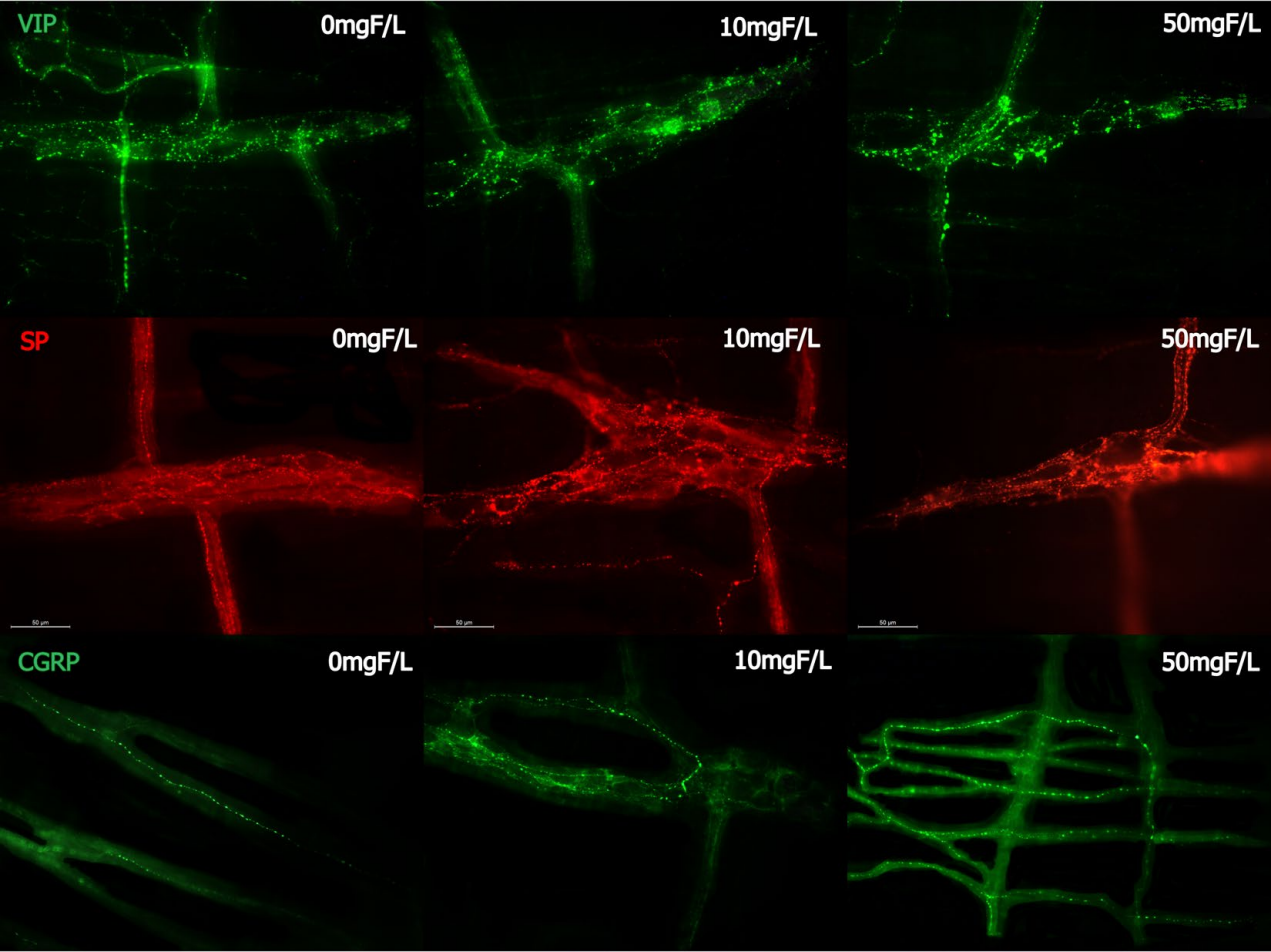
Photomicrography of myenteric varicosities of the rats jejunum after F chronic exposure (0, 10 or 50 mgF/L) for VIP-IR, SP-IR CGRP-IR. 40x Objective.

Typical images of the immunofluorescences are shown in Figs 1 and 2.

### Proteomic analysis of the jejunum

The total numbers of proteins identified in the control, 10 and 50 mgF/L groups were 294, 343 and 322, respectively. These proteins were present in the 3 pooled samples for each group. Among them, 81 (Table S1), 120 (Table S2) and 99 (Table S3) proteins were uniquely identified in the control, 10 mgF/L and 50 mgF/L groups, respectively. In the quantitative analysis of the 10 mgF/L *vs*. control group, 30 proteins with change in expression were detected (Table S4). As for the comparison 50 mgF/L *vs*. control group, 40 proteins with change in expression were found (Table S5). Most of the proteins with changed expression were upregulated in the groups treated with F when compared with the control group (21 and 23 proteins in the groups treated with 10 mgF/L and 50 mgF/L, Tables S4 and S5, respectively).

**Figure 3:**
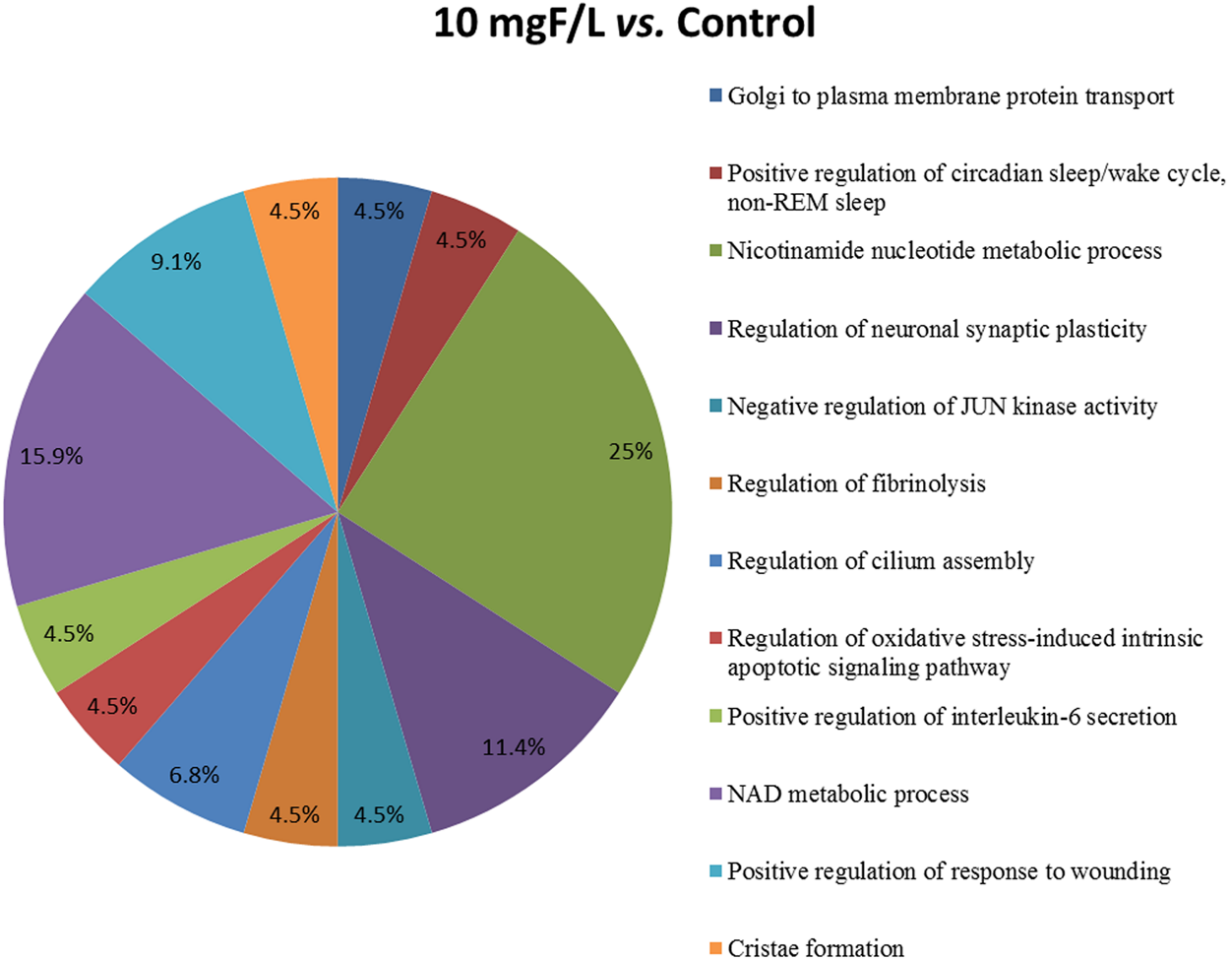
Functional distribution of proteins identified with differential expression in the jejunum of rats exposed to the chronic dose of 10 mgF/L *vs*. Control Group (0 mgF/L). Categories of proteins based on GO annotation Biological Process. Terms significant (Kappa = 0.04) and distribution according to percentage of number of genes association. Proteins access number was provided by UNIPROT. The gene ontology was evaluated according to ClueGo® pluggins of Cytoscape® software 3.4.0^89,90^.

**Figure 4:**
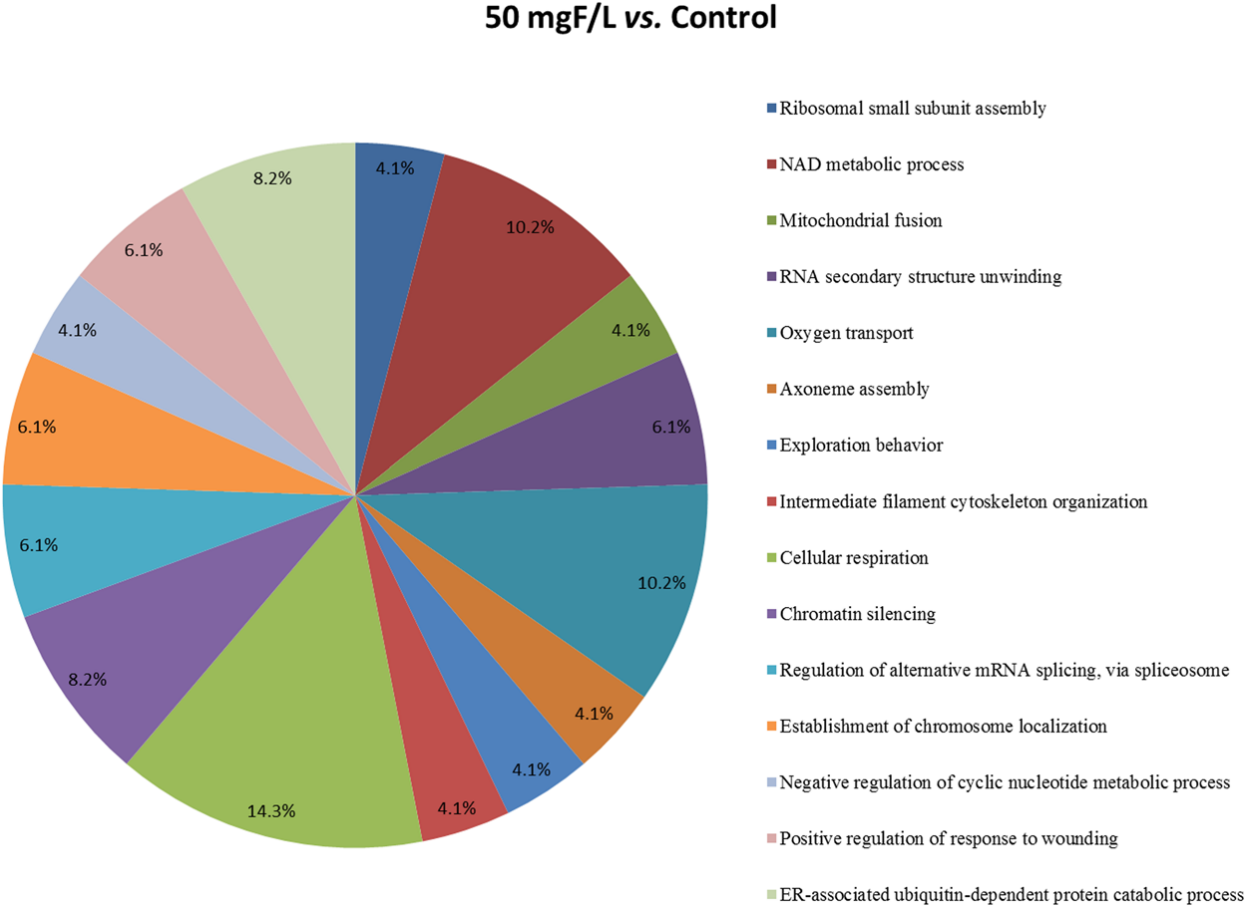
Functional distribution of proteins identified with differential expression in the jejunum of rats exposed to the chronic dose of 50 mgF/L *vs*. Control Group (0 mgF/L). Categories of proteins based on GO annotation Biological Process. Terms significant (Kappa = 0.04) and distribution according to percentage of number of genes association. Proteins access number was provided by UNIPROT. The gene ontology was evaluated according to ClueGo® pluggins of Cytoscape® software 3.4.0^89,90^.

Figures 3 and 4 show the functional classification according to the biological process with the most significant term, for the comparisons 10 mgF/L *vs*. control and 50 mgF/L *vs*. control, respectively. The group exposed to the highest F concentration had the largest alteration, with change in 15 functional categories (Fig. 4). Among them, the categories with the highest percentage of associated genes were: Cellular respiration (14.3%), NAD metabolic process (10.2%), Oxygen transport (10.2%), Chromatin silencing (8.2%) and ER-associated ubiquitin-dependent protein catabolic process (8.2%). Exposure to the lowest F concentration influenced 12 functional categories (Fig. 3). The biological processes with the highest percentage of affected genes were: Nicotinamide nucleotide metabolic process (25%), Regulation of neuronal synaptic plasticity (11.4%), NAD metabolic process (15.9%) and Positive regulation of response to wounding (9.1%). It should be highlighted that Regulation of oxidative stress-induced intrinsic apoptotic signaling pathway was also identified, with 4.5% of affected genes (4.5%).

**Figure 5:**
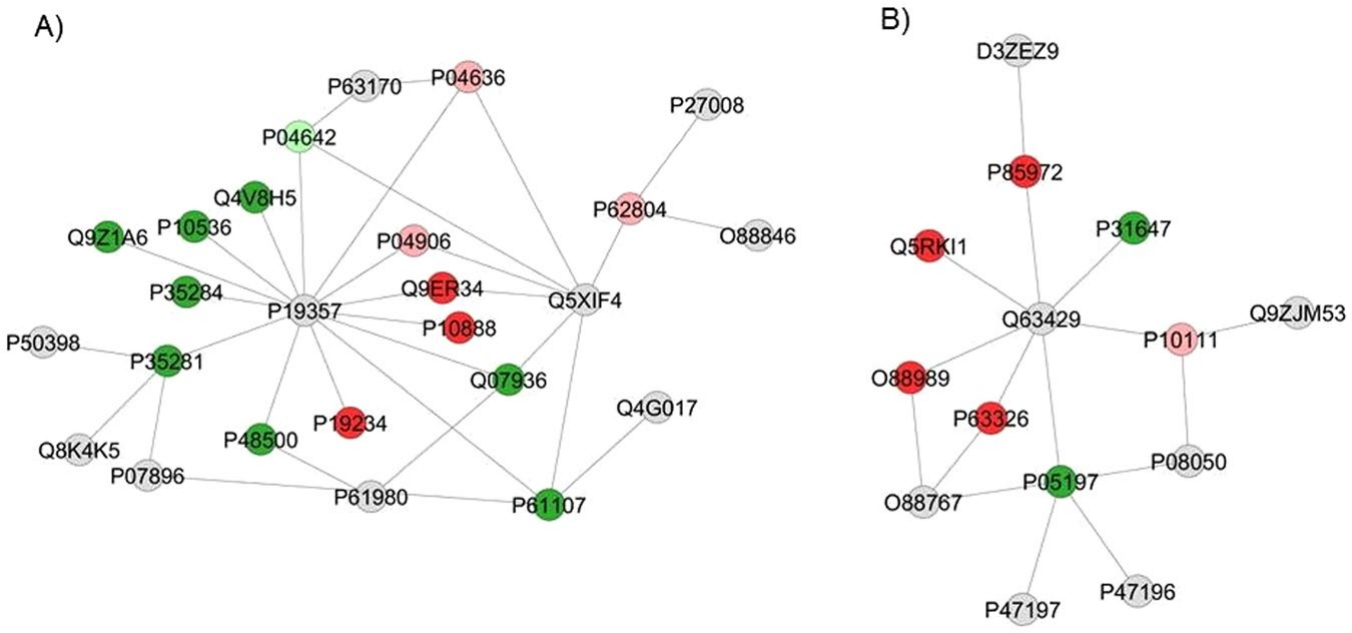
Subnetworks created by ClusterMark® to establish the interaction between proteins identified with differential expression in the 10 mgF/L group in relation to the control group. The color of the nodes indicates the differential expression of the respective named protein with its access code. The dark red and dark green nodes indicate proteins unique to the control and 10 mgF/L groups, respectively. The nodes in gray indicate the interaction proteins that are offered by CYTOSCAPE®, which were not identified in the present study and the light red and light green nodes indicate downregulation and upregulation, respectively. In (**A**), the access numbers in the gray nodes correspond to: *Dynein light chain 1*, *cytoplasmic* (P63170), *Poly [ADP-ribose] polymerase 1* (P27008), *E3 ubiquitin-protein ligase RNF4* (O88846), *Small ubiquitin-related modifier 3* (Q5XIF4), *Nischarin* (Q4G017), *Heterogeneous nuclear ribonucleoprotein K* (P61980), *Peroxisomal bifunctional enzyme* (P07896), *Lethal(2) giant larvae protein homolog 1* (Q8K4K5), *Rab GDP dissociation inhibitor alpha* (P50398) and *Solute carrier family 2*, *facilitated glucose transporter member 4* (P19357). The access numbers of the unique proteins of the control (dark red nodes) correspond to: *Aconitate hydratase*, *mitochondrial* (Q9ER34), *Cytochrome c oxidase subunit 4 isoform 1*, *mitochondrial* (P10888) and *NADH dehydrogenase [ubiquinone] flavoprotein 2*, *mitochondrial* (P19234). The accession numbers of the unique 10 mgF/L (dark green nodes) proteins correspond to: *Aspartyl aminopeptidase* (Q4V8H5), *Ras-related protein Rab-1B* (P10536), *Vigilin* (Q9Z1A6), *Ras-related protein Rab-12* (P35284), *Ras-related protein Rab-10* (P35281), *Triosephosphate isomerase* (P48500), *Annexin A2* (Q07936) and *Ras-related protein Rab-14* (P61107). The access numbers of the downregulated proteins (light red nodes) correspond to: *Malate dehydrogenase*, *mitochondrial* (P04636), *Glutathione S-transferase P* (P04906) and *Histone H4* (P62804). The accession numbers of the upregulated proteins (light green nodes) correspond to: *L-lactate dehydrogenase A chain* (P04642). In (**B)**, the access numbers in the gray nodes correspond to: *Protein Svil* (D3ZEZ9), *Polyubiquitin-C* (Q63429), *Apoptosis-inducing factor 1*, *mitochondrial* (Q9JM53), *Protein deglycase DJ-1* (O88767), *RAC-beta serine/threonine-protein kinase* (P47197), *RAC-alpha serine/threonine-protein kinase* (P47196) and *Gap junction alpha-1 protein* (P08050). The access numbers of the unique proteins of the control (dark red nodes) correspond to: *Vinculin* (P85972), *Eukaryotic initiation factor 4A-II* (Q5RKI1), *Malate dehydrogenase*, *cytoplasmic* (O88989) and *40 S ribosomal protein S10* (P63326). The accession numbers of the single 10 mgF/L (dark green nodes) proteins correspond to: *Elongation factor 2* (P05197) and *Sodium- and chloride-dependent GABA transporter 3* (P31647). The access numbers of the downregulated proteins (light red nodes) correspond to: *Peptidyl-prolyl cis-trans isomerase A* (P10111).

**Figure 6:**
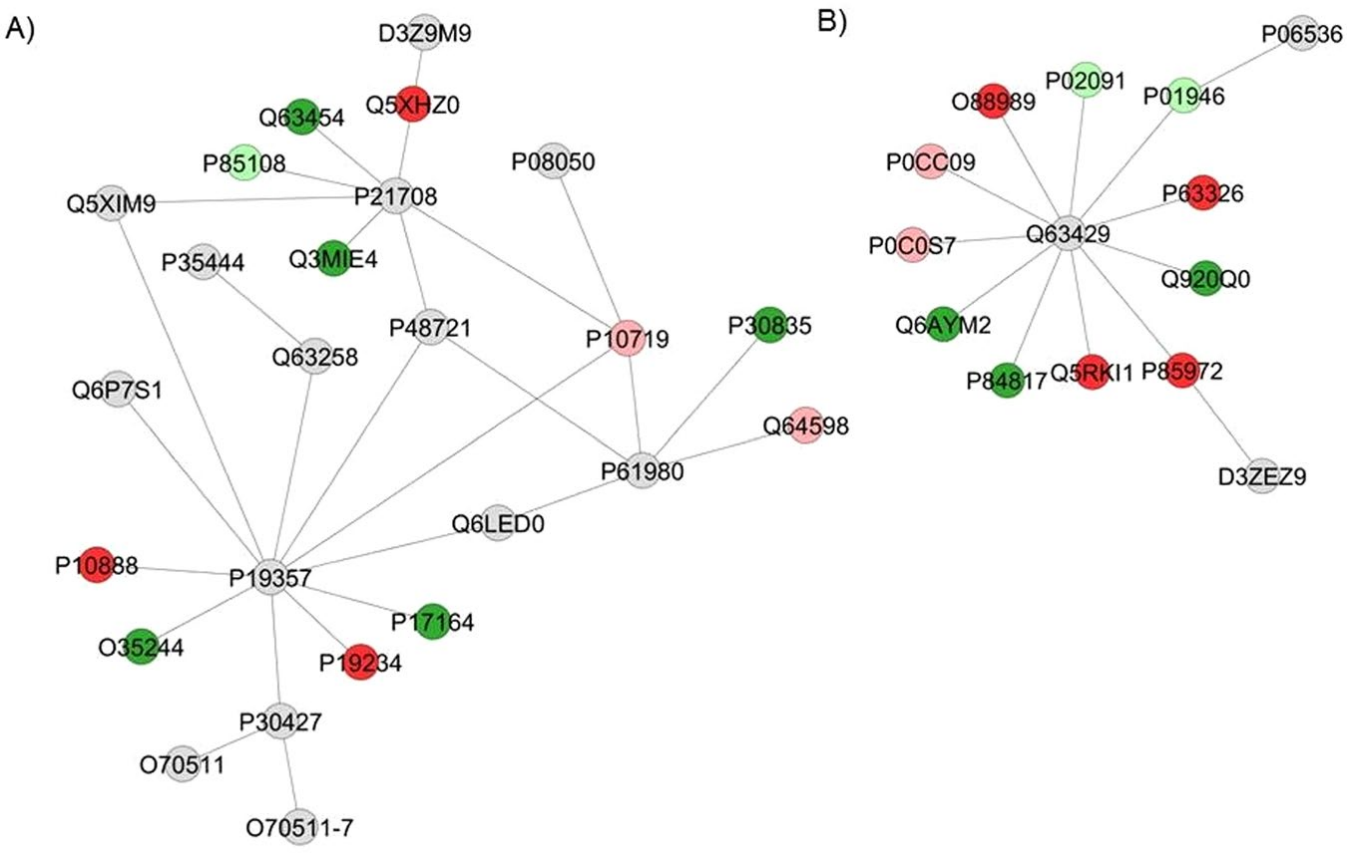
Subnetworks created by ClusterMark® to establish the interaction between proteins identified with differential expression in the 50 mgF/L group in relation to the control group. The color of the nodes indicates the differential expression of the respective named protein with its access code. The dark red and dark green nodes indicate proteins unique to the control and 50 mgF/L groups, respectively. The nodes in gray indicate the interaction proteins that are offered by CYTOSCAPE®, which were not identified in the present study and the light red and light green nodes indicate downregulation and upregulation, respectively. In (**A**), the access numbers in the gray nodes correspond to: *PTEN induced putative kinase 1 (Predicted)* (D3Z9M9), *Mitogen-activated protein kinase 3* (P21708), *T-complex protein 1 subunit beta* (Q5XIM9), *Gap junction alpha-1 protein* (P08050), *Cartilage oligomeric matrix protein* (P35444), *Acid ceramidase* (Q6P7S1), *Integrin alpha-7* (Q63258), *Stress-70 protein*, *mitochondrial* (P48721), *Solute carrier family 2*, *facilitated glucose transporter member 4* (P19357), *Histone H3*.*1* (Q6LED0), *Heterogeneous nuclear ribonucleoprotein K* (P61980), *Ankyrin-3* (O70511), *Plectin* (P30427) and *Ankyrin-3* (O70511-7). The access numbers of the unique proteins of the control (dark red nodes) correspond to: *Malate dehydrogenase*, *cytoplasmic* (O88989), *40 S ribosomal protein S10* (P63326), *Eukaryotic initiation factor 4A-II* (Q5RKI1) and *Vinculin* (P85972). The accession numbers of the unique 50 mgF/L (dark green nodes) proteins correspond to: *Tektin-2* (Q6AYM2), *Mitochondrial fission 1 protein* (P84817) and *Paralemmin-1* (Q920Q0). The access numbers of the downregulated proteins (light red nodes) correspond to: *Histone H2A type 2-A* (P0CC09) and *Histone H2A*.*Z* (P0C0S7). The accession numbers of the upregulated proteins (light green nodes) correspond to: *Hemoglobin subunit beta-1* (P02091) and *Hemoglobin subunit alpha-1/2* (P01946). In (**B**), the access numbers in the gray nodes correspond to: *Polyubiquitin-C (Q63429)*, *Protein Svil (D3ZEZ9) e Glucocorticoid receptor (P06536)*. The access numbers of the unique proteins of the control (dark red nodes) correspond to: *Heat shock protein 75 kDa*, *mitochondrial* (Q5XHZ0), *Cytochrome c oxidase subunit 4 isoform 1*, *mitochondrial* (P10888) and *NADH dehydrogenase [ubiquinone] flavoprotein 2*, *mitochondrial* (P19234). The accession numbers of the single 50 mgF/L (dark green nodes) proteins correspond to: *Mitogen-activated protein kinase 4* (Q63454), *Synaptic vesicle membrane protein VAT-1 homolog* (Q3MIE4), *ATP-dependent 6-phosphofructokinase*, *liver type* (P30835), *Tissue alpha-L-fucosidase* (P17164) and *Peroxiredoxin-6* (O35244). The access numbers of the downregulated proteins (light red nodes) correspond to: *ATP synthase subunit beta*, *mitochondrial* (P10719) and e *Histone H2A type 1-F* (Q64598). The accession numbers of the upregulated proteins (light green nodes) correspond to: *Tubulin beta-2A chain* (P85108).

Figures 5 and 6 show the subnetworks created by CLUSTERMARK for the comparisons 10 mgF/L *vs*. control and 50 mgF/L *vs*. control, respectively. For the 10 mgF/L group (Fig. 5), most of the proteins with change in expression interacted with *Solute carrier family 2*, *facilitated glucose transporter member 4* (GLUT4; P19357) and *Small ubiquitin-related modifier 3* (Q5XIF4) (Fig. 5A) or with *Polyubiquitin-C* (Q63429) and *Elongation factor 2* (P05197) (Fig. 5B). As for the group treated with 50 mgF/L, most of the proteins with change in expression interacted with GLUT4 (P19357) and *Mitogen-activated protein kinase 3* (MAPK3; P21708) (Fig. 6A) or *Polyubiquitin-C* (Q63429) (Fig. 6B).

## Discussion

The small intestine is responsible for absorption of around 70–75% of F^5,21^. As consequence, gastrointestinal symptoms, such as abdominal pain, nausea, vomiting and diarrhea, are the most common occurrence in cases of excessive ingestion of F^22–25^. The mechanisms underlying these changes remain to be determined. Recently, our group took advantage of immunofluorescence and proteomics techniques to evaluate changes in the duodenum of rats after chronic exposure to F^13^. The group treated with 50 mgF/L had a significant decrease in the density of nNOS-IR neurons. Additionally, important morphological changes were seen in HUC/D-IR and nNOS-IR neurons, as well as in VIP-IR, CGRP-IR, and SP-IR varicosities for the groups treated with both 10 and 50 mgF/L. Moreover, profound proteomic alterations were observed in both treated groups. In the group treated with 10 mgF/L, most of the proteins with altered expression were upregulated. On the other hand, downregulation of several proteins was found in the group treated with the highest F concentration^13^.

Many proteins observed in the previous study were correlated with the neurotransmission process, which is essential for the function of the GIT through ENS control. For example, the pattern of intestinal smooth muscle contraction can be modified when the release of neurotransmitters stimulating muscle contraction, such as SP^26^ is increased or when the release of neurotransmitters promoting muscle relaxation, such as NO^27^, is decreased. In the present study, both conditions might have occurred, because we found a significance increase and decrease in the mean values of the SP varicosities area and the density of nNOS-IR neurons, respectively (Table 1), which is in accordance with our previous findings for the duodenum^13^. This finding can be also associated with the significant decrease in the density of HUC/D-IR neurons (Table 1), and it could contribute to the intestinal discomfort and symptoms, such as abdominal pain and diarrhea, observed upon excessive exposure to F.

Another important neurotransmitter that also participates in the control of intestinal motility is VIP. In our study, it was observed a statistically significant increase in the mean value of the areas of VIP-IR myenteric varicosities in the 50 mgF/L group when compared with control. This finding is similar to what was observed in our previous study where duodenum was analyzed^13^ and confirms that this dose of F can compromise the vipergic innervation of the small intestine. For the inhibitory control of motility, the main neurotransmitters involved are NO and VIP^28^, so basically any changes in the vipergic innervation can alter the intestinal motility, leading to a decrease in the tone of the intestinal smooth muscle, which could trigger diarrhea or even increased susceptibility to intestinal infections by decreased intestinal transit^29^. We can also suggest, in this case, that this increase may mean upregulation in the expression of the VIP, as a response to F toxicity since other processes such as axotomy and blocking of axonal transport or hypertrophic alterations promote upregulation of VIP in enteric neurons^30^. This increase can also be related to a neuroprotective role of VIP, because it acts as a potent anti-inflammatory molecule and presents an important antioxidant activity^31–33^. In addition to this, VIP is one of the most important elements involved in enteric neuroplasticity^33^, which is the ENS ability to adapt to any change in its microenvironment^34^. Due to the morphological changes that we observed in our study in the vipergic varicosities, we can suggest that F can induce important neuroplastic changes in the GIT.

Since alterations in the morphology of the intestinal wall infer important pathophysiological processes, we analyzed the total thickness of the intestinal wall, as well as the tunica muscularis separately. The group treated with 50 mgF/L presented a significant decrease in the total thickness of the intestinal wall and an increase in the thickness of the *tunica muscularis*, indicating that F can alter morphologically the jejunum wall. The finding for the *tunica muscularis* of the jejunum is in-line with our previous findings for the duodenum^13^, despite the total thickness of the duodenum wall was not altered. Changes in the number and morphology of myenteric cell bodies may be related to variations of the *tunica muscularis* thickness, which presents the structures responsible for the maintenance, development and plasticity of these neurons^35^. Similar increase in the thickness of the intestinal wall and *tunica muscularis* have been reported in the duodenum and jejunum of rats fed with a high fat diet for 8 weeks, where morphological alterations in the general population of enteric neurons and in the nitrergic population were also detected^36^, emphasizing that intestinal physiology comprises many interconnected mechanisms.

As in our study F caused morphological alterations in different enteric neuronal subtypes, which present several neurotransmitters involved in the GIT motility, it is possible that these alterations affect the GIT function, and promote the important symptomatology of F toxicity on the GIT, such as abdominal pain and diarrhea. We also believe that our results are quite relevant regarding the ENS, since mechanisms of neurodegeneration associated to enteric neuropathies are characterized basically by alterations, damage or loss of enteric neurons, as observed in several important pathologies^37^ and also in our study. Thus, in order to better investigate these findings involving the enteric innervation, we performed the proteomic analysis.

The proteomic approach revealed for both F doses that the majority of the proteins presenting changed expression interacted with *Solute carrier family 2*, *facilitated glucose transporter member 4* (GLUT4) (P19357) and *Polyubiquitin C* (Q63429). In the network comparing 10 mgF/L *vs*. control groups, 17 members of the Ras-related Rab proteins (isoforms 1A, 1B, 3A, 3C, 3D, 4A, 4B, 5A, 8A, 8B, 10, 12, 14, 26, 35, 37, and 43) were uniquely found in the group treated with 10 mgF/L (Table S2), despite some not being present in the network. The GTPases Rab proteins are known as key regulators of intracellular membrane trafficking, from the formation of transport vesicles to their fusion with membranes. Rabs modulate between an inactive form (GDP-bound) and an active form (GTP-bound). The latter can attract to membranes distinct downstream effectors that will lead to vesicle formation, movement, tethering and fusion (UNIPROT). Generally, many studies report Rab proteins as molecules present in the CNS and their specific roles. Although marked differences distinguish the neuronal function between the ENS and CNS, their similarities allow the use of some principles established for the brain environment to be reapplied in the enteric context^38^. Several cellular processes can be altered and promote the enteric neuronal alterations caused by F effects through mechanisms involving the Rab proteins, which are considered neuronal regulators involved in the traffic and signaling of different molecules that promote neurons homeostasis, such as the neurotrophins family of growth factors. The neurotrophins-receptors complexes trigger important signaling pathways that promote development, survival and other neuronal functions through intracellular transport mechanisms mediate by the Rab proteins^39^.

Rab 1A is a regulator of specific vesicular trafficking from the ER to Golgi complex, and in dopaminergic neurons its expression presents a protective effect enhancing the control of motor function in surviving neurons of hemiparkinsonian animals^40^. From the family of the Rab 3 proteins, 3 members were present in the 10 mgF/L group, Rab 3A, Rab 3C, and Rab 3D. The Rab 3 family is observed in different cell types with high exocytic function^41^, in which they function as exocytosis regulators^42^ correlated with neuronal traffic^39^, and are present in synaptic vesicles, modulating the neurotransmitter release^42^. Rab 3A is the most abundant isoform in the brain, where it presents a modulatory function in synaptic membrane fusion through a Ca^2+^-dependent manner^43^. In the peripheral nervous system Rab 3A has increased expression in sciatic nerve lesion area associated to an increase in the expression of two other important proteins that contribute to neurotransmission, synaptophysin and synapsin I^44^. Rab 3C is highly expressed in primary hippocampal neurons, mediating regulated exocytosis^45^, while Rab 3D is present in secretory granules and vesicles of other cell types, such as adipocytes, exocrine glands, hematopoietic cells^46^, and low levels of expression were already identified in the duodenum, confirming its presence in exocrine cells of the GIT^47^.

The Rabs 4A and 4B were also identified as exclusive for the 10 mgF/L, and Rab4 is described as a regulator of early endosomes in the synapses, contributing to neurotransmitter receptor recycling through endosomes acting associated to other molecules in the later steps of the endocytic recycling pathway in dendrites, directing the neuronal membrane receptor trafficking^48^. This process is extremely important for the delivery of neurotransmitter receptors into the synaptic membrane, determining the synaptic function and plasticity. Rab 5A presents a role in axonal and dendritic endocytosis, contributing to the biogenesis of synaptic vesicles^49^. Rab 8 presents the same role as Rab 4, being required to direct into synapses neurotransmitter receptors as the AMPA-type glutamatergic receptors, presenting an important role in the control of synaptic function and plasticity at the postsynaptic membrane^50^.

Rab 10 is required for the secretion of neuropeptides through the release of dense core vesicles, which is a mechanism that modulates neuronal activity^51^. It is also a regulator of membrane trafficking during dendrite morphogenesis, and loss of RAB 10 decreases proximal dendritic arborization in the multi-dendritic PVD neurons^52^. In the CNS Rab 12 is colocalized with M98K, and overexpression of the latter induces cell death in retinal glial cells, while knockdown of Rab 12 reduces M98K-induced cell death in the same cells through the autophagy mechanism^53^.

Rab 26 promotes in the brain the formation of clusters of vesicles in neuritis^54^, and the authors suggest a new mechanism for degradation of synaptic vesicles in which Rab 26 selectively conducts synaptic and secretory vesicles into preautophagosomal structures. In neuronal immortalized cells, Rab 35 promotes neurite differentiation and favors axon elongation in rat primary neurons in an activity-dependent manner^55^.

The fact that several members of the Rab proteins were expressed exclusively in the 10 mgF/L group might indicate that this F concentration could affect the neuronal functions, since different Rab proteins regulate distinct processes in the neuronal environment. Since the 10 mgF/L concentration caused a decrease in the enteric neuronal density, which can compromise the enteric neuronal activity, the expression of several Rab proteins can reflect an attempt to keep the neurotransmission unaltered in the presence of F. Besides the neuronal activity, other important biological mechanisms involve the Rab proteins action. In the network comparing 10 mgF/L *vs*. control groups, the isoforms 1B, 10, 12 and 14 interact with GLUT4, and especially Rab 10 and Rab 14, are required in GLUT4 translocation to the plasma membrane^56,57^. Their increased expression might help to explain the increased sensitivity to insulin recently reported to occur in rats with diabetes induced by streptozotocin exposed to 10 mgF/L in the drinking water^58^. The increased expression of Rab 10 and Rab 14 might facilitate glucose uptake. Rab 37 and Rab 3A, also present among the proteins exclusively expressed in the 10 mgF/L group, are involved in the insulin release. Rab 3A has an important role in the hormone release from pancreatic β-cells with a regulatory control on insulin-containing secretion^59^. Rab 37, with a high sequence homology with Rab 3A, has also been reported to participate in regulated secretion in mammalian cells in the control of insulin exocytosis through a different mechanism of Rab 3A^60^. According to the authors, impairment of Rab 37 expression may contribute to abnormal insulin release in pre-diabetic and diabetic conditions. We can infer that the expression of both proteins indicates that the insulin release mechanism could be altered with this F dose. We also observed an increase in *L-lactate dehydrogenase A chain* (LDH) (P04642) upon exposure to 10 mgF/L. This enzyme converts pyruvate to lactate with regeneration of NADH into NAD^+^. It is an alternative way to supply the lack of oxygen for aerobic oxidation of pyruvate and NADH produced in glycolysis^61^. In fact, the categories nicotinamide nucleotide metabolic process and NAD metabolic process were among the ones with the highest percentage of affected genes when the 10 mgF/L group was compared with control. Previous studies have reported increase in the LDH activity in the serum of infants who consumed water containing more than 2 mgF/L^62^. It was also overexpressed in the brain of rats treated with F^63^. When pyruvate is converted into lactate by LDH, less pyruvate is available to enter into the mitochondria and form acetyl-CoA, which is consistent with the reduction of *Malate dehydrogenase*, *mitochondrial* (P04636) and of enzymes related to the oxidative phosphorylation, such as *Cytochrome c oxidase subunit 4 isoform 1*, *mitochondrial* (P10888) and *NADH dehydrogenase [ubiquinone] flavoprotein 2*, *mitochondrial* (P19234). According to Barbier, *et al*.^3^, F has an inhibitory effect on the activity of citric acid cycle enzymes, in agreement with our finding of reduction in *Malate dehydrogenase*, *mitochondrial*. Another protein with altered expression (downregulation) in the group treated with 10 mgF/L that interacts with GLUT4 was *Glutathione S-transferase P* (P04906) that was also found downregulated in the duodenum of rats treated with the same dose of F^13^. This enzyme is involved in the metabolism and detoxification of xenobiotics^64^. Many proteins with altered expression in the network comparing 10 mgF/L *vs*. control groups interact with *Polyubiquitin C* (Q63429), a highly conserved polypeptide that is covalently bound to other cellular proteins to signal processes such as protein degradation, protein/protein interaction and protein intracellular trafficking^65^. Among them are proteins related to translation, that were absent in the group treated with 10 mgF/L, such as *Eukaryotic initiation factor 4A-II* (Q5RKI1) and *40 S ribosomal protein S10* (P63326). The latter was also reduced in the group treated with 50 mgF/L both in the present study and in a previous study where duodenum was analyzed^13^. In addition, *Peptidyl-prolyl cis-trans isomerase A* (P10111) was reduced in the group treated with 10 mgF/L compared to control, which might impair protein folding. Also involved in protein synthesis, *Elongation factor 2* (P05197) presented altered expression upon exposure to 10 mgF/L. This protein was present only in the group treated with 10 mgF/L, and catalyzes the GTP-dependent ribosomal translocation step during translation elongation (UNIPROT). Differences in expression of all these proteins indicate alterations in distinct steps of protein synthesis upon exposure to 10 mgF/L. Changes in protein synthesis might help to explain the alterations in the thickness of the jejunum wall observed in this group. Interestingly, *Elongation factor 2* interacted with two of the 3 isoforms of the protein kinase AKT, namely *RAC-alpha serine/threonine-protein kinase* (AKT1; P47196) and *RAC-beta serine/threonine-protein kinase* (AKT2; P47197) that mediate protein synthesis and glucose metabolism^66^.

In the network comparing the 50 mgF/L *vs*. control groups (Fig. 6), some proteins with relevance for the neuronal homeostasis were expressed uniquely in the 50 mgF/L, such as *Tektin-2* (Q6AYM2), *Perforin-1* (Q5FVS5), and *Mitochondrial fission 1 protein* (*Fis1*-P84817). The Tektins family has significant expression in adult brain and in embryonic stages of the choroid plexus, the forming retina, and olfactory receptor neurons, and can be considered a molecular target for the comprehension of neural development^67^. Although not present in the subnetwork, Perforin participates in the CD8^+^ T cells response, promoting granule cytotoxicity leading to a fast cellular necrosis of the target cell in minutes^68^ or apoptosis in a period of hours through a mechanism in which the target cell collaborates with perforin to deliver granzymes into the cytosol^69^. Using these mechanisms perforin-dependent, CD8^+^ T cells promote neuronal damage in inflammatory CNS disorders^70^.

Mitochondrial fission is implicated in the cell death through a pathway that involves caspase activation^71^, and *Mitochondrial fission 1 protein* (Fis1) is considered essential for mitochondrial fission^72^. Overexpression of Fis1 caused increase of mitochondrial fragmentation, which conducted to apoptosis or triggered autophagy^73,74^, and neuroprotective effects are correlated with inhibition of Fis1^75^.

The fact that these proteins presented increased expression in relation to the control group can reflect F neurotoxicity on the ENS with the concentration of 50 mgF/L, and could result in the decrease in the density of the general population of neurons since these 3 proteins are involved in pathways that conduct to cell death by distinct mechanisms.

Other proteins with altered expression interacted mainly with GLUT4 (P19357) and *Polyubiquitin C* (Q63429), which was also observed for the network comparing the 10 mgF/L *vs*. control groups (Fig. 5). In addition, *Mitogen-activated protein kinase 3* (MAPK3; P21708) was also an interacting partner as in the duodenum of rats treated with the same concentration of F in the drinking water^13^. Among the proteins that interacted with GLUT4, *Peroxiredoxin-6* (O35244) was present only in the group treated with 50 mgF/L, when compared with control (Fig. 6). This enzyme, located in the cytoplasm, protects cells against oxidative stress, in addition to modulating intracellular signaling pathways. Peroxiredoxins catalyze the reduction of H_2_O_2_ and hydroxyperoxide in water and alcohol^76^. Thus, changes in these proteins expression could be linked to fluoride-induced oxidative stress that has been extensively described in the literature^3,77–81^. In the group treated with 50 mgF/L, there was a remarkable downregulation in several isoforms of Histones, in comparison with control (Fig. 6 and Table S5). The major role described for histones is DNA “packaging”, however, it is also well described that these proteins confer variations in chromatin structure to ensure dynamic processes of transcriptional regulation in eukaryotes^82^. Epigenetic modifications of DNA and histones are fundamental mechanisms by which neurons adapt their transcriptional response to developmental and environmental factors. Modifications in the chromatin of neurons contribute dramatically to changes in the neuronal circuits, and it is possible that histone activity is involved in disorders that compromise neuronal function^83^. Thus, changes in the expression of histones might have contributed to the alterations found in the morphology of enteric neurons in response to F exposure. In addition, structural muscle proteins such as different isoforms of actin and myosin were increased or exclusive in the group treated with 50 mgF/L (Tables S3 and S5), which helps to explain the increase in the thickness of the jejunum *tunica muscularis*.

Probably the most remarkable finding of the present study was that when the groups treated with 10 and 50 mgF/L are compared with control, some proteins related to energetic metabolism presented similar alterations in expression, regardless the dose of F, such as: *Cytochrome c oxidase subunit 4 isoform 1*, *mitochondrial* (P10888), *NADH dehydrogenase [ubiquinone] flavoprotein 2*, *mitochondrial* (P19234), *Malate dehydrogenase*, *mitochondrial* (P04636), *Malate dehydrogenase*, *cytoplasmic* (O88989) and *L-lactate dehydrogenase A chain* (P04642). The absence of *Malate dehydrogenase*, *mitochondrial* (P04636), *Malate dehydrogenase*, *cytoplasmic* (O88989), that form oxaloacetate, absence of *NADH dehydrogenase [ubiquinone] flavoprotein 2*, *mitochondrial* (P19234) that transfers electrons from NADH to respiratory chain in both groups treated with F, as well as the reduction of *ATP synthase subunit beta*, *mitochondrial* (P10719) (only in the group treated with the highest F dose), as well as the increase in *L-lactate dehydrogenase A chain* (P04642) in both groups treated with F indicate an increase in anaerobic metabolism in attempt to obtain energy, since aerobic metabolism is impaired in the presence of F. However, the rate of production of ATP through anaerobic pathways is much lower than that of aerobic pathways, which is in-line with the reports of reduction in the production of ATP induced by exposure to high F doses^3,84^. It is important to highlight that these changes in the expression of proteins associated to energy metabolism induced by exposure to 10 and 50 mgF/L in the drinking water are more pronounced than those observed previously in other organs exposed to roughly the same doses of F^58,63,80,85–88^. This might be due to the fact that the small intestine is responsible for the absorption of around 75% of ingested F^5^, which makes the cells of the intestinal wall exposed to higher doses of F than the cells from the other organs.

In conclusion, chronic exposure to F, especially to the highest concentration evaluated, increased the thickness of the *tunica muscularis* and altered the pattern of protein expression. Extensive downregulation of several isoforms of histones might have contributed to the alterations found in the morphology of enteric neurons in response to F exposure. Additionally, changes in proteins involved in energy metabolism indicate a shift from aerobic to anaerobic metabolism upon exposure to the highest F concentration. These findings provide new insights into the mechanisms involved in F toxicity in the intestine.

## Electronic supplementary material

Supplementary Information

## References

[1] Yan X (2011). Fluoride induces apoptosis and alters collagen I expression in rat osteoblasts. Toxicol Lett.

[2] Buzalaf MA, Pessan JP, Honorio HM, ten Cate JM (2011). Mechanisms of action of fluoride for caries control. Monogr Oral Sci.

[3] Barbier O, Arreola-Mendoza L, Del Razo LM (2010). Molecular mechanisms of fluoride toxicity. Chem Biol Interact.

[4] Whitford GM, Pashley DH (1984). Fluoride absorption: the influence of gastric acidity. Calcif Tissue Int.

[5] Nopakun J, Messer HH, Voller V (1989). Fluoride absorption from the gastrointestinal tract of rats. J Nutr.

[6] Zheng Y, Wu J, Ng JC, Wang G, Lian W (2002). The absorption and excretion of fluoride and arsenic in humans. Toxicol Lett.

[7] Susheela A, Kumar A, Bhatnagar M, Bahadur R (1993). Prevalence of endemic Fluorosis with gastrointestinal manifestations in People living in some North-Indian villages. Fluoride.

[8] Susheela A (1992). Fluoride ingestion and its correlation with gastrointestinal discomfort. Fluoride.

[9] Sharma JD, Jain P, Sohu D (2009). Gastric Discomforts from Fluoride in Drinking Water in Sanganer Tehsil, Rajasthan, India. Fluoride.

[10] Das TK, Susheela AK, Gupta IP, Dasarathy S, Tandon RK (1994). Toxic Effects of Chronic Fluoride Ingestion on the Upper Gastrointestinal-Tract. J Clin Gastroenterol.

[11] Furness, J. B. A comprehensive overview of all aspects of the enteric nervous system. *The Enteric Nervous System*. (Blackwell, 2006).

[12] Sand E (2014). Structural and functional consequences of buserelin-induced enteric neuropathy in rat. BMC Gastroenterol.

[13] Melo CGS (2017). Enteric innervation combined with proteomics for the evaluation of the effects of chronic fluoride exposure on the duodenum of rats. Sci Rep.

[14] Guyton, A. C. & Hall, J. E. *Textbook of medical physiology*. 13 edn, (Elsevier Health Sciences, 2015).

[15] Dunipace AJ (1995). Effect of aging on animal response to chronic fluoride exposure. J Dent Res.

[16] Bradford MM (1976). A rapid and sensitive method for the quantitation of microgram quantities of protein utilizing the principle of protein-dye binding. Anal Biochem.

[17] Lima Leite A (2014). Proteomic analysis of gastrocnemius muscle in rats with streptozotocin-induced diabetes and chronically exposed to fluoride. PLoS One.

[18] Bauer-Mehren A (2013). Integration of genomic information with biological networks using Cytoscape. Methods Mol Biol.

[19] Millan PP (2013). Visualization and analysis of biological networks. Methods Mol Biol.

[20] Orchard S (2012). Molecular interaction databases. Proteomics.

[21] Nopakun J, Messer HH (1990). Mechanism of fluoride absorption from the rat small intestine. Nutr Res.

[22] Vogt RL, Witherell L, LaRue D, Klaucke DN (1982). Acute fluoride poisoning associated with an on-site fluoridator in a Vermont elementary school. Am J Public Health.

[23] Augenstein WL (1991). Fluoride ingestion in children: a review of 87 cases. Pediatrics.

[24] Gessner BD, Beller M, Middaugh JP, Whitford GM (1994). Acute fluoride poisoning from a public water system. N Engl J Med.

[25] Akiniwa K (1997). Re-examination of acute toxicity of fluoride. Fluoride.

[26] Holzer P, Lippe IT (1984). Substance P can contract the longitudinal muscle of the guinea-pig small intestine by releasing intracellular calcium. Brit J Pharmacol.

[27] Rivera LR, Poole DP, Thacker M, Furness JB (2011). The involvement of nitric oxide synthase neurons in enteric neuropathies. Neurogastroenterol Motil.

[28] Benarroch EE (2007). Enteric nervous system: functional organization and neurologic implications. Neurology.

[29] Defani MA, Zanoni JN, Natali MR, Bazotte RB, de Miranda-Neto MH (2003). Effect of acetyl-L-carnitine on VIP-ergic neurons in the jejunum submucous plexus of diabetic rats. Arq Neuropsiquiatr.

[30] Ekelund KM, Ekblad E (1999). Structural, neuronal, and functional adaptive changes in atrophic rat ileum. Gut.

[31] Hermes-Uliana C (2014). Is L-glutathione more effective than L-glutamine in preventing enteric diabetic neuropathy?. Dig Dis Sci.

[32] Veit AP, Zanoni JN (2012). Age-related changes in myosin-V myenteric neurons, CGRP and VIP immunoreactivity in the ileum of rats supplemented with ascorbic acid. Histol Histopathol.

[33] Ekblad E, Bauer AJ (2004). Role of vasoactive intestinal peptide and inflammatory mediators in enteric neuronal plasticity. Neurogastroenterol Motil.

[34] Oste M (2005). The intestinal trophic response to enteral food is reduced in parenterally fed preterm pigs and is associated with more nitrergic neurons. J Nutr.

[35] Gabella, G. In *Physiology of the* Gastrointestinal Tract. (ed L. R. JOHNSON) 335–381 (Raven Press, 1987).

[36] Soares A, Beraldi EJ, Ferreira PE, Bazotte RB, Buttow NC (2015). Intestinal and neuronal myenteric adaptations in the small intestine induced by a high-fat diet in mice. BMC Gastroenterol.

[37] De Giorgio R (2004). New insights into human enteric neuropathies. Neurogastroenterol Motil.

[38] Gershon MD, Ratcliffe EM (2004). Developmental biology of the enteric nervous system: pathogenesis of Hirschsprung’s disease and other congenital dysmotilities. Semin Pediatr Surg.

[39] Bucci C, Alifano P, Cogli L (2014). The role of rab proteins in neuronal cells and in the trafficking of neurotrophin receptors. Membranes (Basel).

[40] Coune PG, Bensadoun JC, Aebischer P, Schneider BL (2011). Rab1A over-expression prevents Golgi apparatus fragmentation and partially corrects motor deficits in an alpha-synuclein based rat model of Parkinson’s disease. J Parkinsons Dis.

[41] Lledo PM (1994). Rab3 proteins: key players in the control of exocytosis. Trends Neurosci.

[42] Schlüter OM, Schmitz F, Jahn R, Rosenmund C, Südhof TC (2004). A complete genetic analysis of neuronal Rab3 function. J Neurosci.

[43] Geppert M, Goda Y, Stevens CF, Südhof TC (1997). The small GTP-binding protein Rab3A regulates a late step in synaptic vesicle fusion. Nature.

[44] Li JY, Jahn R, Dahlström A (1995). Rab3a, a small GTP-binding protein, undergoes fast anterograde transport but not retrograde transport in neurons. Eur J Cell Biol.

[45] van Vlijmen T (2008). A unique residue in rab3c determines the interaction with novel binding protein Zwint-1. FEBS Lett.

[46] Pavlos NJ (2005). Rab3D regulates a novel vesicular trafficking pathway that is required for osteoclastic bone resorption. Mol Cell Biol.

[47] Valentijn JA, van Weeren L, Ultee A, Koster AJ (2007). Novel localization of Rab3D in rat intestinal goblet cells and Brunner’s gland acinar cells suggests a role in early Golgi trafficking. Am J Physiol Gastrointest Liver Physiol.

[48] Hoogenraad CC (2010). Neuron specific Rab4 effector GRASP-1 coordinates membrane specialization and maturation of recycling endosomes. PLoS Biol.

[49] de Hoop MJ (1994). The involvement of the small GTP-binding protein Rab5a in neuronal endocytosis. Neuron.

[50] Gerges NZ, Backos DS, Esteban JA (2004). Local control of AMPA receptor trafficking at the postsynaptic terminal by a small GTPase of the Rab family. J Biol Chem.

[51] Sasidharan N (2012). RAB-5 and RAB-10 cooperate to regulate neuropeptide release in Caenorhabditis elegans. Proc Natl Acad Sci USA.

[52] Zou W, Yadav S, DeVault L, Nung Jan Y, Sherwood DR (2015). RAB-10-Dependent Membrane Transport Is Required for Dendrite Arborization. PLoS Genet.

[53] Sirohi K (2013). M98K-OPTN induces transferrin receptor degradation and RAB12-mediated autophagic death in retinal ganglion cells. Autophagy.

[54] Binotti B (2015). The GTPase Rab26 links synaptic vesicles to the autophagy pathway. Elife.

[55] Villarroel-Campos D (2016). Rab35 Functions in Axon Elongation Are Regulated by P53-Related Protein Kinase in a Mechanism That Involves Rab35 Protein Degradation and the Microtubule-Associated Protein 1B. J Neurosci.

[56] Sano H (2007). Rab10, a target of the AS160 Rab GAP, is required for insulin-stimulated translocation of GLUT4 to the adipocyte plasma membrane. Cell metabolism.

[57] Brewer PD, Habtemichael EN, Romenskaia I, Coster AC, Mastick CC (2016). Rab14 limits the sorting of Glut4 from endosomes into insulin-sensitive regulated secretory compartments in adipocytes. The Biochemical J.

[58] Lobo JG (2015). Low-Level Fluoride Exposure Increases Insulin Sensitivity in Experimental Diabetes. J Dent Res.

[59] Lang J (1999). Molecular mechanisms and regulation of insulin exocytosis as a paradigm of endocrine secretion. Eur J Biochem.

[60] Ljubicic S (2013). The GTPase Rab37 Participates in the Control of Insulin Exocytosis. PLoS One.

[61] Lehninger, A. L., Nelson, D. L. & Cox, M. M. *Lehninger principles of biochemistry*. 3. ed. edn, 975 (2002).

[62] Xiong X (2007). Dose-effect relationship between drinking water fluoride levels and damage to liver and kidney functions in children. Environ Res.

[63] Ge Y, Niu R, Zhang J, Wang J (2011). Proteomic analysis of brain proteins of rats exposed to high fluoride and low iodine. Arch Toxicol.

[64] Kaminsky LS, Zhang QY (2003). The small intestine as a xenobiotic-metabolizing organ. Drug Metab Dispos.

[65] Ciechanover A, Schwartz AL (1998). The ubiquitin-proteasome pathway: the complexity and myriad functions of proteins death. Proc Natl Acad Sci USA.

[66] Bottermann K, Reinartz M, Barsoum M, Kotter S, Godecke A (2013). Systematic Analysis Reveals Elongation Factor 2 and alpha-Enolase as Novel Interaction Partners of AKT2. PLoS One.

[67] Norrander J, Larsson M, Ståhl S, Höög C, Linck R (1998). Expression of ciliary tektins in brain and sensory development. J Neurosci.

[68] Waterhouse NJ (2006). Cytotoxic T lymphocyte-induced killing in the absence of granzymes A and B is unique and distinct from both apoptosis and perforin-dependent lysis. J Cell Biol.

[69] Pipkin ME, Lieberman J (2007). Delivering the kiss of death: progress on understanding how perforin works. Curr Opin Immunol.

[70] Meuth SG (2009). Cytotoxic CD8+ T cell-neuron interactions: perforin-dependent electrical silencing precedes but is not causally linked to neuronal cell death. J Neurosci.

[71] Perfettini JL, Roumier T, Kroemer G (2005). Mitochondrial fusion and fission in the control of apoptosis. Trends Cell Biol.

[72] Chen H, Chan DC (2005). Emerging functions of mammalian mitochondrial fusion and fission. Hum Mol Genet 14 Spec No..

[73] James DI, Parone PA, Mattenberger Y, Martinou JC (2003). hFis1, a novel component of the mammalian mitochondrial fission machinery. J Biol Chem.

[74] Gomes LC, Scorrano L (2008). High levels of Fis1, a pro-fission mitochondrial protein, trigger autophagy. Biochim Biophys Acta.

[75] Wang Y (2017). Neuroprotective Effect and Mechanism of Thiazolidinedione on Dopaminergic Neurons *In Vivo* and *In Vitro* in Parkinson’s Disease. PPAR Res.

[76] Hofmann B, Hecht HJ, Flohe L (2002). Peroxiredoxins. Biol Chem.

[77] Shanthakumari D, Srinivasalu S, Subramanian S (2004). Effect of fluoride intoxication on lipidperoxidation and antioxidant status in experimental rats. Toxicology.

[78] Stirnimann G, Kessebohm K, Lauterburg B (2010). Liver injury caused by drugs: an update. Swiss Med Wkly.

[79] Zhan XA, Wang M, Xu ZR, Li WF, Li JX (2006). Effects of fluoride on hepatic antioxidant system and transcription of Cu/Zn SOD gene in young pigs. J Trace Elem Med Biol.

[80] Pereira HA (2013). Proteomic analysis of liver in rats chronically exposed to fluoride. PLoS One.

[81] Pereira HA (2016). Fluoride Intensifies Hypercaloric Diet-Induced ER Oxidative Stress and Alters Lipid Metabolism. PLoS One.

[82] Maze I, Noh KM, Soshnev AA, Allis CD (2014). Every amino acid matters: essential contributions of histone variants to mammalian development and disease. Nat Rev Genet.

[83] Riccio A (2010). Dynamic epigenetic regulation in neurons: enzymes, stimuli and signaling pathways. Nat Neurosci.

[84] Strunecka A, Patocka J, Blaylock R, Chinoy N (2007). Fluoride interactions: from molecules to disease. Current Signal Transduction Therapy.

[85] Xu H (2005). Proteomic analysis of kidney in fluoride-treated rat. Toxicol Lett.

[86] Kobayashi CA (2009). Proteomic analysis of kidney in rats chronically exposed to fluoride. Chem Biol Interact.

[87] Niu R (2014). Proteomic analysis of hippocampus in offspring male mice exposed to fluoride and lead. Biol Trace Elem Res.

[88] Carvalho JG (2013). Renal proteome in mice with different susceptibilities to fluorosis. PLoS One.

[89] Bindea G, Galon J, Mlecnik B (2013). CluePedia Cytoscape plugin: pathway insights using integrated experimental and in silico data. Bioinformatics.

[90] Bindea G (2009). ClueGO: a Cytoscape plug-in to decipher functionally grouped gene ontology and pathway annotation networks. Bioinformatics.

