# Supplementary material for: Chronic treatment with fluoride affects the jejunum: insights from proteomics and enteric innervation analysis
Source: Sci Rep. 2018 Feb 16;8:3180. doi: 10.1038/s41598-018-21533-4 (PMC5816638; doi:10.1038/s41598-018-21533-4)
Supplement: Supplementary file 1 — Supplementary Information [file 41598_2018_21533_MOESM1_ESM.pdf]

Title

Chronic treatment with fluoride affects the jejunum: insights from proteomics and enteric innervation analysis

Aline Salgado Dionizio [1]

Carina Guimarães Souza Melo [1]

Isabela Tomazini Sabino-Arias [1]

Talita Mendes Silva Ventura [1]

Aline Lima Leite [1]

Sara Raquel Garcia Souza [2]

Erika Xavier Santos [2]

Alessandro Domingues Heubel[1]

Juliana Gadelha Souza [1]

Juliana Vanessa Colombo Martins Perles[2]

Jacqueline Nelisis Zanoni [2]

Marília Afonso Rabelo Buzalaf [1*]

[1] Department of Biological Sciences, Bauru School of Dentistry, University of São Paulo, Bauru, Brazil.

[2] Department of Morphophysiological Sciences, State University of Maringá, Maringá, Brazil.

[*] **Corresponding author**

Marília Afonso Rabelo Buzalaf

Alameda Octávio Pinheiro Brisolla, 9-75

Bauru-SP, Brazil, 17012-901

Phone: # 55 14 3235-8346

Email: mbuzalaf@fob.usp.br

Supplementary Table S1. Proteins identified exclusively in the jejunum of rats of control group.

[a]Access number	Protein name	*PLGS Score*
P63326	40S ribosomal protein S10	86.29
Q9ER34	Aconitate hydratase, mitochondrial	56.82
Q68FP8	Adenylate kinase 8	57.06
O09178	AMP deaminase 3	63.74
Q7TP90	Arrestin domain-containing protein 4	119.76
D3ZTV7	Ash2 (Absent, small, or homeotic)-like (Drosophila) (Predicted)	58.22
Q8K1M8	BMP/retinoic acid-inducible neural-specific protein 2	77.73
Q6AXW0	Borealin	85.75
Q6MGA9	Bromodomain-containing protein 2	36.96
Q5XIR8	Clathrin heavy chain linker domain-containing protein 1	45.92
A6JUQ6	Clavesin-2	125.81
Q6AY97	Coiled-coil domain-containing protein 91	59.96
F1LQC8	Cyclin-dependent kinase 7	106.02
P51952	Cyclin-dependent kinase 7 (Fragment)	106.02
P00173	Cytochrome b5	110.98
P10888	Cytochrome c oxidase subunit 4 isoform 1, mitochondrial	95.86
Q5PPJ4	Deoxyhypusine hydroxylase	97.36
Q9Z1Z3	Epsin-2	77.85
Q5RKI1	Eukaryotic initiation factor 4A-II	82.70
F1LM27	Gamma-aminobutyric acid receptor subunit pi	73.39
F8WFK6	Glutathione peroxidase	101.68
G3V6A8	Golgi autoantigen, golgin subfamily b, macrogolgin 1, isoform CRA_c	34.84
Q5XHZ0	Heat shock protein 75 kDa, mitochondrial	386.33
D3ZMT4	Histidine decarboxylase	60.20
M0RCB8	Histone H3 (Fragment)	61.07
Q4KLJ2	Integral membrane protein 2A	45.35
G3V667	Integrin, alpha 6, isoform CRA_a	42.01
Q63679	Lysine-specific demethylase 3A	37.26
O88989	Malate dehydrogenase, cytoplasmic	152.62
Q5EB94	Myocardial zonula adherens protein	63.78
P19234	NADH dehydrogenase [ubiquinone] flavoprotein 2, mitochondrial	85.87
A0JPJ0	Nicotinamide nucleotide adenylyltransferase 1	157.53
Q78PB6	Nuclear distribution protein nudE-like 1	64.22
F1LUD2	Olfactory receptor	75.58
G3V7X0	Outer dense fiber of sperm tails 2, isoform CRA_e	52.20
Q63468	Phosphoribosyl pyrophosphate synthase-associated protein 1	40.67
P20961	Plasminogen activator inhibitor 1	110.43
P30427	Plectin	66.57
Q99PT0	Probable ATP-dependent RNA helicase DDX52	66.23
P10960	Prosaposin	101.41
D4A332	Protein Ankle1	67.55
D3ZUL3	Protein Col6a1	115.75
D4AD15	Protein Eif4g1	39.23
D3ZXM4	Protein Evi5l	163.14

D3Z8Z2	Protein Fam53b	41.90
D3ZQI9	Protein Iffo1	51.19
D4AE58	Protein Kank1	33.30
Q6IFW7	Protein Krt28	46.34
F1MAF7	Protein Krt33b	63.25
D3Z9W1	Protein LOC100271845	93.32
D3ZJY5	Protein LOC100360905	81.87
D4A609	Protein LOC100361741	53.06
D3ZET2	Protein LOC100910851	39.52
M0RC68	Protein LOC100911797	39.52
D3ZMU9	Protein LOC102547078 (Fragment)	92.54
D4A4U8	Protein LOC299277	110.61
D3ZBD0	Protein Msl1	78.45
F1M0Q9	Protein Pm20d1 (Fragment)	74.24
D4A404	Protein Psd3	91.46
D3Z8R4	Protein Rbm25l1	116.19
D3ZW64	Protein RGD1560556	43.61
D3ZH53	Protein RGD1561871	56.93
F1LT36	Protein RGD1564698	86.29
F1LVT5	Protein Rundc1	42.69
D4A4R7	Protein Serpina1f	70.53
M0R5B1	Protein Shisa8	78.09
D4A3B0	Protein Tln2	47.51
D3ZE22	Protein Ttll3	46.14
D4A7F0	Protein Ubr3 (Fragment)	26.72
P85973	Purine nucleoside phosphorylase	111.91
D3ZXK9	Purine nucleoside phosphorylase (Fragment)	111.91
Q3B7T9	Rab11 family-interacting protein 1	65.46
P49797	Regulator of G-protein signaling 3	58.82
G3V9Q2	Regulator of G-protein signalling 3, isoform CRA_b	58.82
Q9R095	Sperm flagellar protein 2	51.95
D3ZTA9	Sulfotransferase	59.56
Q4V8I3	Tensin-4	41.40
F1LYK6	tRNA (guanine(37)-N1)-methyltransferase	60.45
P0CD94	Ubiquinol-cytochrome-c reductase complex assembly factor 3	153.05
P85972	Vinculin	96.17
Q5MYW4	Zinc finger protein 667	43.27

Identified proteins are organized according to the alphabetical order of proteins. The ID is based on protein ID from the UniProt protein database (http://www.uniprot.org/).

Supplementary Table S2. Proteins identified exclusively in the jejunum of rats chronically exposed to water containing 10 mgF/L.

[a]Access number	Protein name	*PLGS Score*
Q6AY33	Acrosin-binding protein	56.20
P38918	Aflatoxin B1 aldehyde reductase member 3	153.75
Q6PCU3	Aldoc protein	68.15
Q9JJH9	Alpha-2u globulin	53.82
D3ZVB9	Ankyrin repeat domain 23 (Predicted), isoform CRA_a	104.93
D3ZCC5	Ankyrin repeat domain 24 (Predicted), isoform CRA_d	59.06
Q07936	Annexin A2	117.91
Q4V8H5	Aspartyl aminopeptidase	90.11
Q8R4G8	BTB/POZ domain-containing protein KCTD1	60.79
O35783	Calumenin	127.44
Q66HA5	Coiled-coil and C2 domain-containing protein 1A	44.93
G3V927	Discs, large homolog-associated protein 4 (Drosophila)	52.25
P97839	Disks large-associated protein 4	54.65
Q63572	Dual specificity testis-specific protein kinase 1	95.42
Q64610	Ectonucleotide pyrophosphatase/phosphodiesterase family member 2	85.33
P05197	Elongation factor 2	55.56
M0RBF8	Exocyst complex component 4	60.85
P09117	Fructose-bisphosphate aldolase C	95.90
Q5I287	Germ cell-less homolog 1 (Drosophila), isoform CRA_a	60.01
B5DEZ6	Glucosamine-6-phosphate isomerase	97.01
Q04807	Glycosylation-dependent cell adhesion molecule 1	151.29
Q63942	GTP-binding protein Rab-3D	72.18
Q3KRF2	High density lipoprotein binding protein (Vigilin)	39.09
G3V6G1	Immunoglobulin joining chain	105.29
Q920F3	KH domain-containing, RNA-binding, signal transduction-associated protein 2	56.02
Q5U2S4	Leucine rich repeat containing 2	59.37
Q8R5M3	Leucine-rich repeat-containing protein 15	35.57
M0R476	Metabotropic glutamate receptor 1	35.57
P31424	Metabotropic glutamate receptor 5	48.36
F1MAQ5	Microtubule-associated protein	26.44
P15146	Microtubule-associated protein 2	26.44
P02688	Myelin basic protein	87.34
G3V6P7	Myosin, heavy polypeptide 9, non-muscle	35.64
Q62812	Myosin-9	35.64
P97738	Neuronal pentraxin-2	59.16
Q2YDU6	Nuclear prelamin A recognition factor	61.54
D3ZLY6	Olfactory receptor	121.70
P0CAX5	Oligophrenin-1	36.60
P00481	Ornithine carbamoyltransferase, mitochondrial	93.90
F1LRE5	Oxysterol-binding protein	37.41
D3ZLH5	Plexin-B3	41.08
O08628	Procollagen C-endopeptidase enhancer 1	80.57
D4AAT1	Protein Adamts8	60.31
D3ZZ20	Protein Afg3l1	58.65
F1LN92	Protein Afg3l2	50.73
D4A7K0	Protein Tmem242	144.96
D4AD05	Protein Crocc	32.25
Q5XI02	Protein disulfide-isomerase-like protein of the testis	37.97

D3ZRE8	Protein Efcc1	70.94
M0R5H1	Protein Etl4 (Fragment)	44.63
D3ZX40	Protein Fam65c	47.87
F1LSX0	Protein Gzmbl2 (Fragment)	83.25
Q6IFV5	Protein Krt36	65.67
G3V6H0	Protein LOC100363782	72.18
F1LWE4	Protein LOC100910977	72.21
M0R620	Protein LOC100912565	270.84
F1M1G2	Protein Maneal	47.13
D3ZV75	Protein Mfsd1	71.46
D4AB60	Protein Mtbp	38.64
D3ZFU9	Protein Mylk	46.23
M0R915	Protein Naip6	47.42
D3Z8Y9	Protein Pnma3	88.38
D4A0G7	Protein Rab37	72.18
F1M8F0	Protein Rbm7	65.13
B2RYC3	Protein RGD1306746	53.17
F7FM32	Protein RGD1311345	78.42
D3ZYC4	Protein RGD1563680	112.27
D3ZN86	Protein RGD1565323	147.87
B5DFL9	Protein Sestd1	75.09
D4AC81	Protein Slc51a	59.38
F1M2T7	Protein Srrm4	49.73
D3ZAF7	Protein Tbc1d2b	51.81
B5DFD6	Protein Tie1	36.14
D4AA88	Protein Tp73	50.65
Q9EPJ1	Protein Twist1	112.96
F1M8H2	Protein Wars2	61.46
A0A096MIV6	Protein Wbscr17	54.48
B2RYB0	Protein Wdr25l	53.51
D4A365	Protein Zbtb40	42.10
Q5RKJ9	RAB10, member RAS oncogene family	72.18
Q62796	RalA-binding protein 1	50.87
Q9Z1C8	Rap guanine nucleotide exchange factor 3	41.18
P35281	Ras-related protein Rab-10	72.18
P35284	Ras-related protein Rab-12	72.18
P61107	Ras-related protein Rab-14	72.18
Q6NYB7	Ras-related protein Rab-1A	72.18
P10536	Ras-related protein Rab-1B	72.18
P51156	Ras-related protein Rab-26	72.18
Q5U316	Ras-related protein Rab-35	72.18
P63012	Ras-related protein Rab-3A	72.18
P62824	Ras-related protein Rab-3C	72.18
Q53B90	Ras-related protein Rab-43	72.18
P05714	Ras-related protein Rab-4A	72.18
P51146	Ras-related protein Rab-4B	72.18
M0RC99	Ras-related protein Rab-5A	84.73
P35280	Ras-related protein Rab-8A	72.18
P70550	Ras-related protein Rab-8B	72.18
D4AAM1	RCG48016, isoform CRA_c	395.50
Q5FVT1	RCG55460, isoform CRA_a	50.87
P81128	Rho GTPase-activating protein 35	69.97
R9PXY2	RIB43A domain with coiled-coils 2, isoform CRA_a	54.87
P20793	Serine/threonine-protein kinase MAK	64.55
P36394	Sex-determining region Y protein (Fragment)	63.97

D3ZED8	Protein Pmel	46.91
P31647	Sodium- and chloride-dependent GABA transporter 3	56.87
O35814	Stress-induced-phosphoprotein 1	115.58
G3V8I4	Syntaxin 4A (Placental), isoform CRA_a	52.26
Q08850	Syntaxin-4	52.26
Q68FW7	Threonine--tRNA ligase, mitochondrial	51.32
E9PTD9	Toll-like receptor	53.37
O08950	Transcription initiation factor IIA subunit 2	126.89
F1M7T6	Translocon-associated protein subunit gamma	154.77
Q03191	Trefoil factor 3	140.71
P48500	Triosephosphate isomerase	87.60
Q7TNK6	tRNA (guanine(10)-N2)-methyltransferase homolog	68.47
P63149	Ubiquitin-conjugating enzyme E2 B	117.61
Q5RJN9	Uncharacterized protein C14orf79 homolog	168.06
Q9Z1A6	Vigilin	44.45
Q71RJ2	Voltage-dependent calcium channel gamma-2 subunit	114.44
Q5U2Y0	WD repeat domain phosphoinositide-interacting protein 4	65.00

Identified proteins are organized according to the alphabetical order of proteins. The ID is based on protein ID from the UniProt protein database (http://www.uniprot.org/).

Supplementary Table S3. Proteins identified exclusively in the jejunum of rats chronically exposed to water containing 50 mgF/L.

[a]Access number	**Protein name**	***PLGS Score***
Q63570	26S protease regulatory subunit 6B	97.66
P62198	26S protease regulatory subunit 8	61.88
Q6AYY8	Acetyl-coenzyme A transporter 1	56.97
Q6P7S1	Acid ceramidase	66.82
Q5U301	A-kinase anchor protein 2	118.01
D4ADM6	Alkaline phosphatase	55.07
Q63910	Alpha globin	59.39
G3V7W7	Aminopeptidase N	64.03
P15684	Aminopeptidase N	64.03
Q62901	Arginine-glutamic acid dipeptide repeats protein	26.94
B5DEX9	Arid3a protein	60.04
D3ZAF6	ATP synthase subunit f, mitochondrial	145.66
D3ZCM3	ATP-binding cassette, sub-family G (WHITE), member 4	73.10
P30835	ATP-dependent 6-phosphofructokinase, liver type	60.62
D3ZXQ0	Carboxylic ester hydrolase	42.22
Q9WTT2	Caseinolytic peptidase B protein homolog	29.83
F1LMT2	Centlein	48.71
Q3B7T8	Centrosomal protein of 44 kDa	77.36
Q68EJ0	Cytochrome b5 reductase 4	46.55
D3Z7Y1	Cytochrome P450 2C7 (Fragment)	49.22
D3ZH41	Cytoskeleton-associated protein 4 (Predicted)	75.43
Q5M9G8	DDB1- and CUL4-associated factor 11	70.70
P60924	Death ligand signal enhancer	54.09
Q5U2T2	Dehydrodolichyl diphosphate synthase	54.29
Q5RK17	Diablo homolog (Drosophila)	69.43
Q3B8Q2	Eukaryotic initiation factor 4A-III	94.58
F1LMQ2	Farnesyl pyrophosphate synthase	65.62
D4ABB4	F-box/LRR-repeat protein 15	66.59
P43278	Histone H1.0	60.10
D3ZMG5	Hypothetical LOC300207 (Predicted)	90.09
D3ZFH4	Hypothetical LOC314467 (Predicted)	40.80
Q63258	Integrin alpha-7	44.08
D4A6K5	Interleukin 27 receptor, alpha (Predicted)	53.50
B2RYC8	Interleukin-1 receptor-associated kinase 3	65.66
Q6IFW6	Keratin, type I cytoskeletal 10	49.00
Q6IFW8	Keratin, type I cytoskeletal 27	108.44
D3ZIA5	Kinesin-like protein	73.68
Q62813	Limbic system-associated membrane protein	53.88
Q6QI46	LRRGT00162	61.29
P84817	Mitochondrial fission 1 protein	71.56
Q63454	Mitogen-activated protein kinase 4 (Fragment)	53.29
Q9WUJ3	Myomegalin	57.04
D4A3H8	NIMA (Never in mitosis gene a)-related kinase 11 (Predicted)	65.44
G3V8R1	Nucleobindin 2, isoform CRA_b	54.07
B5DFH4	Papss2 protein	121.84
Q920Q0	Paralemmin-1	63.67
Q5FVS5	Perforin 1 (Pore forming protein)	46.18
P35763	Perforin-1	46.18

O35244	Peroxiredoxin-6	87.65
D3ZTP9	Piwi-like protein	47.65
D3ZAN6	Poly(A) polymerase gamma (Predicted)	78.89
D4A8I8	Polymerase (DNA directed), iota (Predicted), isoform CRA_c	60.68
D3ZB30	Polypyrimidine tract binding protein 1, isoform CRA_c	53.14
Q00438	Polypyrimidine tract-binding protein 1	53.14
Q5PQT5	Progestin and adipoQ receptor family member V	55.50
Q4V7A8	Protein ABHD18	55.91
D3ZTI3	Protein Cdh24	32.21
F1M7J7	Protein Cep250 (Fragment)	51.99
O88767	Protein deglycase DJ-1	79.83
F1LT14	Protein Frmd5	69.00
G3V8R3	Protein Hbz	59.39
G3V646	Protein Hsf2bp	50.15
M0R3X5	Protein L1td1	37.55
D4A7Z5	Protein LOC100360940	69.43
G3V8P7	Protein LOC100911794	31.65
M0R983	Protein LOC688320	70.10
F1M9Z7	Protein Lrrc3c	64.06
B0BNB4	Protein Meaf6	59.42
D4A7N2	Protein Mettl10	84.91
D3ZKF3	Protein Morc1	54.30
F1LMD5	Protein Mtf2	108.10
D3ZD36	Protein RGD1306739	41.58
G3V8I7	Protein RGD735029	54.09
D3ZC97	Protein Rgs9bp	66.61
F1LX07	Protein Slc25a12 (Fragment)	65.62
F1LYZ6	Protein Snapc4	65.30
M0R8U7	Protein Spata17 (Fragment)	173.95
D4A9F1	Protein Tango6	40.21
F1LV37	Protein Tnrc6b	28.69
F1LUG6	Protein Ttc24 (Fragment)	38.92
D4ACL2	Protein Ttc38	54.53
D4AA63	Protein Ubqln2	42.89
Q5J3K1	Protein Vom1r62	72.76
D3ZIN6	Protein Zfp27 (Fragment)	91.09
D4A1E1	Protein Zmynd15	52.35
Q921A2	Proton myo-inositol cotransporter	59.04
F1M7Z9	Regulating synaptic membrane exocytosis 4	111.56
Q64578	Sarcoplasmic/endoplasmic reticulum calcium ATPase 1	50.17
Q4FZX7	Signal recognition particle receptor subunit beta	106.18
P48721	Stress-70 protein, mitochondrial	57.24
P23739	Sucrase-isomaltase, intestinal	32.26
Q3MIE4	Synaptic vesicle membrane protein VAT-1 homolog	148.10
Q5XIM9	T-complex protein 1 subunit beta	56.14
Q6AYM2	Tektin-2	103.76
P49430	Thromboxane-A synthase	36.34
P17164	Tissue alpha-L-fucosidase	53.61
D4A556	Uncharacterized protein	55.91
Q3KRF3	Uncharacterized protein C1orf131 homolog	58.30
Q4V8C4	WD repeat-containing protein 5B	69.96

Identified proteins are organized according to the alphabetical order of proteins. The ID is based on protein ID from the UniProt protein database (http://www.uniprot.org/).

Supplementary Table S4. Proteins identified with significantly altered expression in the jejunum of rats treated with 10 mgF/L in the drinking water in comparison to control.

[a]Access number	Protein name	*PLGS Score*	Ratio 10 mg/L F: Control
O70177	Carboxylic ester hydrolase	77.78	1.79
P04642	L-lactate dehydrogenase A chain	185.71	1.65
F1LPR6	Protein Ighm(Fragment)	79.05	1.54
P42123	L-lactate dehydrogenase B chain	59.78	1.52
D4A2K1	Protein Hoga1	87.86	1.45
G3V9Y1	Myosin, heavy polypeptide 10, non-muscle, isoform CRA_b	68.24	1.34
P01946	Hemoglobin subunit alpha-1/2	579.84	1.27
Q63862	Myosin-11 (Fragments)	110.66	1.26
A0A0A0MY09	Endoplasmin	82.66	1.25
F7FK40	Tropomyosin 1, alpha, isoform CRA_c	165.97	1.25
Q64122	Myosin regulatory light polypeptide 9	318.32	1.22
Q66HD0	Endoplasmin	82.66	1.21
P13832	Myosin regulatory light chain RLC-A	205.79	1.20
B0BMS8	Myl9 protein	318.32	1.19
P06685	Sodium/potassium-transporting ATPase subunit alpha-1	100.28	1.19
P18666	Myosin regulatory light chain 12B	205.79	1.17
Q9ESV6	Glyceraldehyde-3-phosphate dehydrogenase, testis-specific	240.77	1.14
P00884	Fructose-bisphosphate aldolase B	391.00	1.13
Q66HT1	Fructose-bisphosphate aldolase	391.00	1.12
P04797	Glyceraldehyde-3-phosphate dehydrogenase	393.6	1.12
D3ZRN3	Protein Actbl2	1720.7	1.06
P04636	Malate dehydrogenase, mitochondrial	1140.94	0.91
P02770	Serum albumin	478.3	0.89
P10111	Peptidyl-prolyl cis-trans isomerase A	567.27	0.82
D3ZJ08	Histone H3	139.14	0.79
P62804	Histone H4	1762.24	0.71
M0R6Y8	Phosphoglycerate kinase	440.19	0.54
P04906	Glutathione S-transferase P	130.58	0.54
Q9ERC0	Neuropeptide Y/peptide YY-Y2 receptor	77.69	0.53
D4A8D5	Filamin, beta (Predicted)	56.51	0.30

Identified proteins are organized according to the ratio score. The ID is based on protein ID from the UniProt protein database (http://www.uniprot.org/).

Supplementary Table S5. Proteins identified with significantly altered expression in the jejunum of rats treated with 50 mgF/L in the drinking water in comparison to control.

[a]Access number	Protein name	*PLGS Score*	Ratio 50 mg/L F: Control
F1LPR6	Protein Ighm (Fragment)	79.05	1.86
G3V741	Phosphate carrier protein, mitochondrial	58.81	1.84
Q00729	Histone H2B type 1-A	113.51	1.79
P01946	Hemoglobin subunit alpha-1/2	579.84	1.57
P04642	L-lactate dehydrogenase A chain	185.71	1.57
Q63862	Myosin-11 (Fragments)	110.66	1.36
Q64122	Myosin regulatory light polypeptide 9	318.32	1.27
P13832	Myosin regulatory light chain RLC-A	205.79	1.25
A0A0A0MY09	Endoplasmin	82.66	1.25
P68370	Tubulin alpha-1A chain	274.37	1.25
B0BMS8	Myl9 protein	318.32	1.23
Q66HD0	Endoplasmin	82.66	1.22
P85108	Tubulin beta-2A chain	453.08	1.22
P18666	Myosin regulatory light chain 12B	205.79	1.21
F7FK40	Tropomyosin 1, alpha, isoform CRA_c	165.97	1.21
P48675	Desmin	222.3	1.15
D4A2K1	Protein Hoga1	87.86	1.14
P02091	Hemoglobin subunit beta-1	1483.69	1.13
P00770	Mast cell protease 2	401.85	1.13
P11517	Hemoglobin subunit beta-2	615.47	1.11
P34058	Heat shock protein HSP 90-beta	202.44	1.11
O88752	Epsilon 1 globin	661.9	1.09
D3ZRN3	Protein Actbl2	1720.70	1.05
P10719	ATP synthase subunit beta, mitochondrial	722.19	0.95
P04636	Malate dehydrogenase, mitochondrial	1140.94	0.93
P02262	Histone H2A type 1	863.45	0.88
Q64598	Histone H2A type 1-F	863.45	0.88
Q4FZT6	Histone H2A type 3	863.45	0.88
P0CC09	Histone H2A type 2-A	863.45	0.87
D3ZXP3	Histone H2A	863.45	0.87
P0C169	Histone H2A type 1-C	863.45	0.87
P0C170	Histone H2A type 1-E	863.45	0.87
Q00728	Histone H2A type 4	863.45	0.87
A9UMV8	Histone H2A.J	863.45	0.87
P0C0S7	Histone H2A.Z	863.45	0.87
P84245	Histone H3.3	139.14	0.66
Q6LED0	Histone H3.1	139.14	0.64
D3ZJ08	Histone H3	139.14	0.63
P62804	Histone H4	176.24	0.61
Q66H84	MAP kinase-activated protein kinase 3	119.36	0.53

Identified proteins are organized according to the ratio score. The ID is based on protein ID from the UniProt protein database (http://www.uniprot.org/).
